# Functional Remodeling of Benign Human Prostatic Tissues *In Vivo* by Spontaneously Immortalized Progenitor and Intermediate Cells

**DOI:** 10.1002/stem.284

**Published:** 2010-01-28

**Authors:** Ming Jiang, Douglas W Strand, Suzanne Fernandez, Yue He, Yajun Yi, Andreas Birbach, Qingchao Qiu, Johannes Schmid, Dean G Tang, Simon W Hayward

**Affiliations:** a Departments of Urological Surgery, Vanderbilt University Medical Center and Vanderbilt-Ingram Cancer CenterNashville, Tennessee, USA; b Departments of Cancer Biology, and, Vanderbilt University Medical Center and Vanderbilt-Ingram Cancer CenterNashville, Tennessee, USA; c Departments of Genetic Medicine, Vanderbilt University Medical Center and Vanderbilt-Ingram Cancer CenterNashville, Tennessee, USA; d Department of Vascular Biology and Thrombosis Research, Center for Biomolecular Medicine and Pharmacology, Medical University of ViennaVienna, Austria; e Department of Carcinogenesis, University of Texas M.D. Anderson Cancer Center, Science Park-Research DivisionSmithville, Texas, USA

**Keywords:** Tissue-specific stem cells, Progenitor cells, Tissue regeneration, Cell transplantation, Self-renewal

## Abstract

Tissue remodeling or regeneration is believed to initiate from multipotent stem and progenitor cells. We report here the establishment of two spontaneously immortalized adult non-tumorigenic human prostate epithelial cell lines, NHPrE1 and BHPrE1. NHPrE1 (CD133^high^/CD44^high^/OCT4^high^/PTEN^high^) was characterized as a putative progenitor cell, and BHPrE1 (p63^high^/p53^high^/p21(WAF1)^high^/RB^high^) was characterized as a putative epithelial intermediate cell. Genomic analysis demonstrated an abnormal karyotype with genomic rearrangements including PTEN amplification in NHPrE1 and CTNNB1 (β-catenin) amplification in BHPrE1 cells. Embedded three-dimensional culture of NHPrE1 showed greater branching than BHPrE1. A tissue recombination-xenografting model was utilized to compare remodeling of human prostatic tissues in vivo. A series of tissue recombinants, made by mixing different ratios of human prostatic epithelial cells and inductive rat urogenital sinus mesenchyme, were grafted to the renal capsule of severe combined immunodeficient mice. Both cell lines were able to regenerate benign secretory ductal-acinar architecture in vivo, containing intact basal and luminal epithelial layers confirmed by the expression of appropriate CK profiles. Prostate-specific antigen, 15-lipoxygenase-2, androgen receptor, and NKX3.1 proteins were appropriately expressed in the regenerated epithelia. Regeneration of benign prostatic glandular structures could be achieved using as few as 10 NHPrE1 cells, whereas 200,000 BHPrE1 cells were required to achieve prostatic architecture. This suggests a greater proportion of progenitor/stem cells in NHPrE1 than in BHPrE1. These cell lines provide important data on progenitor and intermediate cell phenotypes and represent significant new tools for the elucidation of molecular mechanisms of human prostatic regeneration, pathogenesis, and carcinogenesis.

## INTRODUCTION

The human prostate is a canalized ductal-acinar structure that develops from solid cords initiated by the budding of endodermal epithelium of the embryonic urogenital sinus (UGS) [1,2]. These cords grow into the surrounding mesenchyme, then branch and canalize to form a secretory ductal-acinar structure with tall columnar secretory luminal cells and a flattened basal epithelium. Concurrent with epithelial differentiation, the adjacent stroma develops to form a predominantly fibromuscular phenotype [3,4]. Differentiated basal and luminal epithelial cells express appropriate cytokeratins (CK) and biomarkers characteristic of a molecularly definitive lineage commitment. Basal cells express p63 and CKs 5 and 14, whereas luminal cells express CKs 8 and 18 as well as species- and prostate-specific secretory proteins including prostate-specific antigen (PSA) and prostatic acid phosphatase in humans [5]. Prostate cancer (Pca) is characterized by a loss of basal epithelial cells [6]. During development, testicular androgens elicit paracrine interactions between epithelium and stroma in a series of coordinated molecular events, which occurs in a proximal to distal direction in the growing prostate [2,7,8]. The anatomy, histology, and molecular profiling of human prostate have obvious differences from those of rodents [9]. These include the expression of specific proteins, notably PSA and 15-lipoxygenase-2 (15-LOX-2), in human but not rodent prostate [10,11].

The concept of primitive stem and progenitor cells with the capacity for self-renewal and multilineage differentiation has been important in developing a model to understand the molecular mechanisms of normal prostatic development, maturation, and functional homeostasis [5,12]. This idea is also important to comprehend how tissues are remodeled during inflammatory repair or in carcinogenesis resulting from genomic insult, metabolic oxidative stress, or inflammation [13,14]. However, at a practical level there have been few human cell lines available that accurately recapitulate prostatic development and that can be used to examine these concepts. To pursue studies relevant to normal human prostate biology and as a starting point for studies on human disease, there is an urgent need for human prostate epithelial cell lines that show phenotypes that match human tissue samples. Tissue recombination is a valuable tool for the analysis of the functional remodeling of benign human prostatic tissues in an immunodeficient mouse model [9]. While a number of non-tumorigenic immortalized human prostate epithelial (HPrE) cell lines have been established using viral SV-40Tag or E6/E7 infection including BPH-1 [15], and RWPE-1 [16], none of these accurately recapitulate normal human prostatic growth and function. Tissue recombinants generated using BPH-1 cells exhibit solid cords and squamous hyperplastic differentiation [17]. RWPE-1-tissue recombinants show a more differentiated acinus-like structure [18]. These cells can be used to answer specific questions. However, the viral oncogenes seriously impair their relevance to many aspects of human prostatic biology. None of the immortalized lines available accurately recapitulates the well-differentiated mature ductal-acinar system characteristic of human prostate.

In this report, we describe the establishment and characterization of adult, non-tumorigenic human prostate-specific epithelial progenitor and intermediate cell lines. NHPrE1, a spontaneously immortalized “normal” human prostate epithelial cell line, that was established from parent NHPrE0 cells, and BHPrE1, a spontaneously immortalized benign human prostate epithelial cell line from BHPrE0. Cell lines were characterized using immunofluorescence (IF) staining, Western blotting, and array-comparative genomic hybridization (array-CGH) analysis as organ-specific epithelial progenitor and intermediate cell types, respectively. We further demonstrated that these cells differentiate into mature, functional human prostatic ductal-acinar structures in vivo using a tissue recombination-xenografting assay with inductive rat urogenital sinus mesenchyme (UGM). The in vitro characteristics of the cells in relation to putative progenitor and intermediate/transit amplifying cell marker expression are shown to be reflective of their behavior in vivo.

## Materials and Methods

### Primary Culture and Spontaneous Immortalization

HPrE cells (NHP8, designated NHPrE0 here), primary cultured “normal” human prostate epithelial cells isolated from a 41-year-old healthy male prostate, were purchased from Cambrex (Charles City, IA, http://www.cambrex.com) and cultured in the supplied serum-free conditional medium [19]. These cells went into crisis after four serial passages showing classic signs of senescence and apoptosis. We tested different cell culture media and optimized a formulation, here designated “HPrE-conditional medium,” using 50/50 Dulbecco's modified Eagles medium (DMEM)/F12 containing 5% fetal bovine serum (FBS; Atlanta Biologicals, Lawrenceville, GA, http://www.atlantabio.com), 1% insulin-transferrin-selenium-X, ITS (Gibco, Grand Island, NY, http://www.invitrogen.com), 0.4% bovine pituitary extract (BPE; Hammond Cell Tech, Windsor, CA, http://www.hammondcelltech.com), and 1:1,000 10 ng/ml epidermal growth factor (EGF; Sigma-Aldrich, St. Louis, MO, http://www.sigmaaldrich.com) with 1% antibiotic-antimycotic mix (Ab/Am; Life Technologies, Rockville, MD, http://www.lifetech.com), similar to a previously described formulation [20]. This formulation was used for culture, growth, and passaging of NHPrE0 cells. The NHPrE0 cells adapted to the HPrE-conditional culture medium for 3 days and were subsequently passaged five times at the dilution ratio (1:2) per 3 days. At this point a number of colonies composed of small cells grew out and were passaged in excess of 25 times at a suitable dilution ratio (1:2) per 3 days. Two cell lines were established by this method, NHP8-No.1.1 and NHP8-No.1.2. The NHP8-No.1.2 cells, re-designated as NHPrE1, were used for the following experiments, and, after more than 75 subsequent passages, they are considered to be an immortalized line. These cells were routinely passaged in HPrE-conditional medium containing 5% FBS and 1% Ab/Am.

A benign human prostate surgical sample was processed by collagenase hyaluronidase digestion to release epithelial organoids as previously described [20,21]. Organoids were stored in liquid nitrogen. Organoids were recovered and plated on the bottom of a T-25 tissue culture flask and cultured in keratinocyte serum-free medium supplemented with EGF and BPE. These gave rise to primary cultures containing small epithelial-like cells that grew out from the organoids in 3–7 days. Primary cultures of BHPrE0 cells resulted in small cells that were passaged when they reached >75% confluence. After four or five passages, the cells stopped growing, remaining alive but quiescent. The culture medium was changed to the HPrE-conditional medium, and the cells were passaged 10 times at a 1:2 dilution. Small progenitor-like colonies in the primary cultures were identified and trypsinized for collection and enrichment. Cells were passaged in excess of 25 times (1:2) per 3 days and designated BHPrE1. Both NHPrE1 and BHPrE1 have now been maintained in excess of 75 passages with no detected phenotypic changes.

Both NHPrE1 and BHPrE1 cells were retrovirally infected by a pBird–cytomegalovirus–enhanced green fluorescent protein (EGFP) vector [22] and selected by FACS–green fluorescent protein (GFP) sorting. When cultured, these cells were uniformly green on fluorescence microscopy.

### Cellular Viability and Proliferation

NHPrE1 and BHPrE1 cells were seeded at 2,000 cells per well in 24-well plates in the HPrE-conditional medium. Cell proliferation was determined using the Cell Titer 96 assay (Promega, Madison, WI, http://www.promega.com) at indicated time points according to the manufacturer's protocol. After 2 hours of incubation at 37°C, absorbance at 490 nm wavelength was read using a microplate reader.

### Side Population Analysis

NHPrE1 and BHPrE1 cell lines and their parental primary cells NHPrE0 and BHPrE0 were suspended at 1 × 10^6^ cells/ml in prewarmed DMEM plus 5% FBS. Then 5 μg/ml Hoechst 33342 (bis-benzimide, from a 1 mM stock; Sigma-Aldrich) was added, as well as 50 μM verapamil (from a 5 mM stock in ethanol; Sigma-Aldrich) in the + verapamil samples. The cell suspensions were incubated for 120 minutes at 37°C with frequent intermittent mixing. The tubes containing the cell suspensions were placed on ice, and cells were pelleted at 4°C and resuspended in cold PBS with 2% FBS. Propidium iodide (2 μg/ml) was added to discriminate dead cells. Side population was analyzed using 325 nm ultraviolet laser excitation and emission bandpass filters 400/40 nm and 510/20 nm, separated by a dichroic 470-nm longpass filter (LSR I; Becton, Dickinson and Company, Franklin Lakes, NJ, http://www.bd.com).

### Three-Dimensional Culture

For a three-dimensional (3D)-on-top assay, 1,500 NHPrE1 or BHPrE1 cells in 2% growth factor–reduced (GFR) Matrigel (Becton, Dickinson and Company) in HPrE-conditional medium were seeded on a 96-well plate coated with 10 μl GFR Matrigel. Each assay was performed for 9 days, after which structures were fixed and stained with FITC-labeled phalloidin (Sigma-Aldrich) to detect F-actin. For the 3D-embedded assay, 1,500 NHPrE1 or BHPrE1 cells in 100 μl GFR Matrigel were seeded in a 96-well plate (Greiner tissue culture treated, M0687; Sigma-Aldrich) coated with 10 μl GFR Matrigel, and 100 μl HPrE-conditional medium was added once the gel solidified.

### Animals

Adult male severe combined immunodeficient (SCID) mice (C.B-17/IcrHsd-SCID) were purchased (Harlan, Indianapolis, IN, http://www.harlan.com). All work involving animals was performed under protocols reviewed and approved by the Vanderbilt Institutional Animal Care and Use Committee.

### Tissue Recombinants and Subrenal Capsule Xenografting

Rat UGM, the inductive mesenchymal cells surrounding the epithelial core of the urogenital sinus, was prepared from E18.5 embryonic fetuses as previously described [23,24]. To prepare tissue recombinants, rat UGM was mixed with human prostate epithelial cells at different ratios as discussed in the text. The cell mixture was pelleted and resuspended in 50 μl rat-tail collagen (pretitrated to pH 7.4). After polymerization, the collagen was overlaid with growth medium. After incubation overnight at 37°C, the tissue recombinants were grafted under the renal capsule of intact C.B-17/IcrHsd-SCID mouse. Hosts were euthanized at various time points after grafting as noted in Results.

### Antibodies and Reagents

A set of primary antibodies were used in these experiments of immunohistochemical (IHC) and IF staining and Western blotting (supporting information). The secondary antibodies were FITC-conjugated anti-mouse (Sigma-Aldrich) or biotinylated anti-rabbit (DAKO, Glostrup, Denmark, http://www.dako.com). ABC kit was purchased from Vector Laboratories, Burlingame, CA (http://www.vectorlabs.com).

### Hematoxylin and Eosin, IHC, and IF Staining

Tissue recombinants were dissected and fixed in 10% phosphate-buffered formalin overnight, transferred to 50% ethanol, and then processed to paraffin. Samples were sectioned for eight successive layers at 5 μm intervals and stained with hematoxylin and eosin (H&E). IHC and IF were performed as previously described. After deparaffinization with Histoclear (National Diagnostics, Atlanta, GA, http://nationaldiagnostics.com), antigens were unmasked by heating samples in unmasking solution (Vector Laboratories). Endogenous peroxidase activity was quenched using 0.3% hydrogen peroxide and blocked with Clean Vision (ImmunoVision Technologies, Burlingame, CA, http://www.immunovisiontech.com). Samples were then incubated with primary antibodies. After washing in PBS, the slides were incubated in biotinylated secondary antibody for 1 hour. Then the slides were incubated in ABC solution to amplify the signals before visualizing with 3,3′-diaminobenzidine (DAB) and mounting. Human prostate epithelial cells were cultured on slides for 3 days and fixed in 50% methanol/ethanol for 10 min at room temperature and stored in PBS for IF staining.

### Western Blotting

Cells were grown in T-75 flasks for 3 days and lysed with TNN buffer (50 mM Tris-HCl, 150 mM NaCl, and 0.5% NP40 (pH, 7.4)) containing Roche's Complete Proteinase Inhibitor Cocktail Tablets (Roche Diagnostics, Basel, Switzerland, http://www.roche-applied-science.com) after washing with cold PBS. The whole lysates were clarified at 13,000 rpm for 20 min at 4°C. The total protein levels were quantified and samples were stored at −80°C for use. Approximately 50 μg protein/well was loaded and electrophoresed through 10% NuPAGE Bio-Tris gel (Invitrogen) and transferred to nitrocellulose membranes. Membranes were blocked for 1 hour in 1×TBS-T (TBS, 0.1% Tween 20) containing 5% Difco skim milk (Becton, Dickinson and Company), incubated with the primary antibody × TBS-T containing 5% skim milk overnight at 4°C, washed for 30 min in TBS-T, and followed with the horseradish peroxidase-conjugated secondary antibody (1:2,000, Amersham Biosciences, Piscataway, NJ, http://www.amersham.com) diluted in 1×TBS-T containing 5% skim milk for 1 hour at room temperature. Amersham ECL plus detection reagent (GE Healthcare, Pittsburgh, PA, http://www.gehealthcare.com) was used to visualize protein bands. The β-actin was used for the loading control.

### Array-CGH Analysis

Genomic DNA from NHPrE1 and BHPrE1 cell lines and their parental primary cells NHPrE0 and BHPrE0 were extracted with a DNeasy Blood and Tissue kit (Qiagen, Hilden, Germany, http://www1.qiagen.com) according to the manufacturer's instructions. Genomic DNA was fragmented and random-prime labeled as described [25,26] and hybridized to human oligonucleotide microarrays. The oligonucleotide array contains unique 236,000 elements designed for CGH profiling (Human Genome CGH 244A; Agilent Technologies, Palo Alto, CA, http://www.agilent.com). The median interval between mapped elements was 8.9 kb. Fluorescence ratios of scanned images of the arrays were processed to identify statistically significant transitions in copy number by using a circular binary segmentation algorithm [25,26]. In this study, significant copy-number changes were determined on the basis of segmented profiles only. To define ploidy level, array-CGH assay was supplemented with G-band karyotyping. At least 40 DAPI-stained metaphase spreads were evaluated per sample.

### DIGMAP Analysis Method

Chromosome mapping of expression data (differential gene locus mapping (DIGMAP)), which aligns the known chromosomal location of a gene to its expression value deduced by array-CGH analysis, was performed as previously described [27]. The output files from these statistical analyses were processed by the DIGMAP Viewer and differential flag region mapping programs. The DIGMAP Viewer program reads the DIGMAP source file containing CGH data and partitions the CGH data into subsets by chromosome number and subchromosomal locations. A graphical presentation was generated using a heat map to represent each data point with a colored cell that quantitatively reflected the original differential copy number intensities. Genomic regions exhibiting differential gene expression were marked as differential flag regions by visual inspection of the graphical displays.

### Accession Number

All array-CGH data have been deposited at the GEO database (accession number GSE18122).

## RESULTS

### Regeneration of Prostatic Glandular Tissues in Mice by Primary Human Benign Epithelial Cells

**Figure 1:**
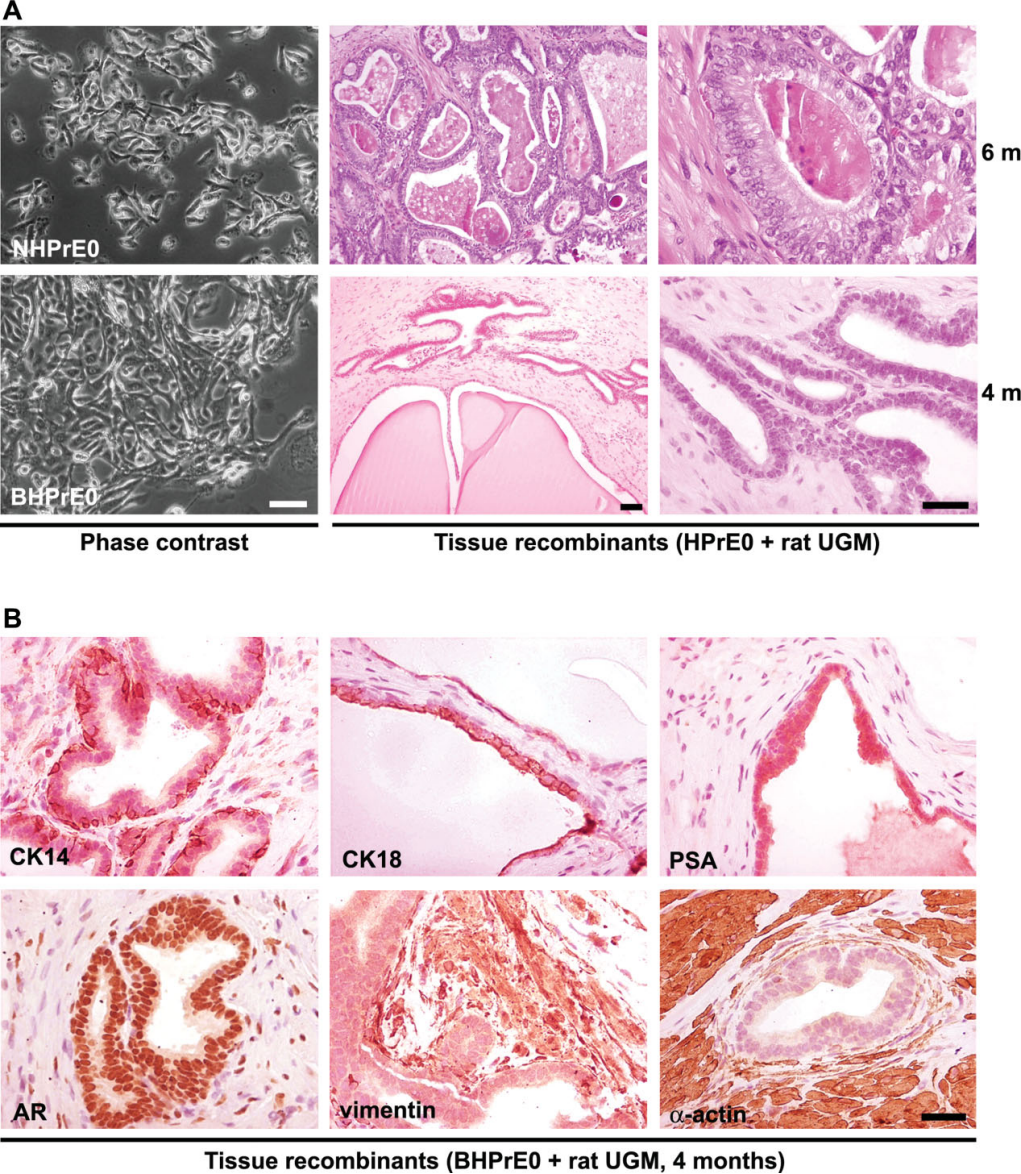
Reconstruction of benign glandular tissues by primary human prostate epithelial cells NHPrE0 and BHPrE0. **A:** Phenotypically, primary HPrE cells NHPrE0 and BHPrE0 under phase contrast microscopy are small with some elongated spindle-shaped cells with limited cobblestone morphology. Scale bar = 100 μm. Hematoxylin and eosin–stained sections of tissue recombinants made using NHPrE0 or BHPrE0 plus rat UGM show well-differentiated glandular formation at 6 months or 4 months after grafting, respectively. The glandular structure is composed of two epithelial layers, luminal and basal. Secretion is found in the lumen of the gland. Scale bar = 100 μm (low magnification) or 50 μm (high magification). **B:** Immunophenotyping of regenerated human prostatic epithelium plus rat UGM. In the ductal-acinar structure, CK14 protein is expressed in basal cells, whereas CK18 protein is localized in secretory luminal cells. PSA is positive in the luminal cells lining differentiated ducts. All epithelial and some stromal cells show AR immunoreactivity. Vimentin is positive in some periacinar stroma. α-actin identifies the smooth muscle layer. Scale bar = 50 μm. Abbreviations: AR, androgen receptors; BHPrE0, primary cultured benign human prostate epithelial cells isolated from a prostate surgical sample; HPrE, human prostate epithelial cells; NHPrE0 (NHP8), primary cultured “normal” human prostate epithelial cells isolated from a donated, 41-year-old, healthy, male prostate; PSA, prostate-specific antigen; UGM, urogenital sinus mesenchyme.

To investigate whether cultures of human prostatic epithelial cells at early passages can regenerate functional glandular structure in vivo, we utilized two strains of cells, NHPrE0 (NHP8) and BHPrE0 cells, which were isolated from a benign prostate surgical sample (Fig. 1A). These cells were used to generate tissue recombinants with inductive rat UGM, which were xenografted into immunocompromised mouse hosts. NHPrE0 is a mixed population of epithelial cells. The majority of such cells were characterized as basal in vitro, with few luminal-type cells [19]. BHPrE0 cells were morphologically similar to the NHPrE0 cells, with more luminal-type cells detected with CK18 IF staining (data not shown). In tissue recombinants, well-differentiated and apparently mature ductal-acinar architecture was found. The number of such structures was dependent upon the ratio of implanted epithelial cells to rat UGM and on the postgrafting time course. Glandular prostatic structures were found in tissue recombinants of NHPrE0 cells by 6 months after grafting [19] and with BHPrE0 cells by 4 months (Fig. 1A). A set of biomarkers and IHC staining showed protein expression patterns in the tissue recombinants made using BHPrE0 cells (Fig. 1B). These results were similar to NHPrE0-tissue recombinants [19]. Ku70-staining (data not shown), which distinguishes human cells by strong nuclear immunoreactivity, was strongly positive in all epithelial cells. CK14 (Fig. 1B) and p63 (data not shown) were strongly expressed in the basal cells. CK18 (Fig. 1B) and CK8 (data not shown) were detected in the luminal cells. Androgen receptors (AR) were expressed in luminal and basal epithelial cells and in some stromal cells (Fig. 1B). PSA (Fig. 1B), which is a distinctive human prostate-specific marker, was expressed in the mature luminal epithelial cells appropriate to the observed differentiation status. These results demonstrate that benign, non-tumorigenic human prostate tissue architecture can be generated in mice from isolated primary cultures of epithelial cells using recombination with rat UGM. This is consistent with previous observations using primary digests of human prostatic epithelial organoids [9].

### Generation and Characterization of Human Prostatic Epithelial Progenitor and Intermediate Cell Lines by Spontaneous Immortalization

Two immortalized human prostatic epithelial cell lines designated NHPrE1 and BHPrE1 were generated as described in Materials and Methods. These cells were derived from the parental NHPrE0 and BHPrE0 strains, respectively.

**Figure 2:**
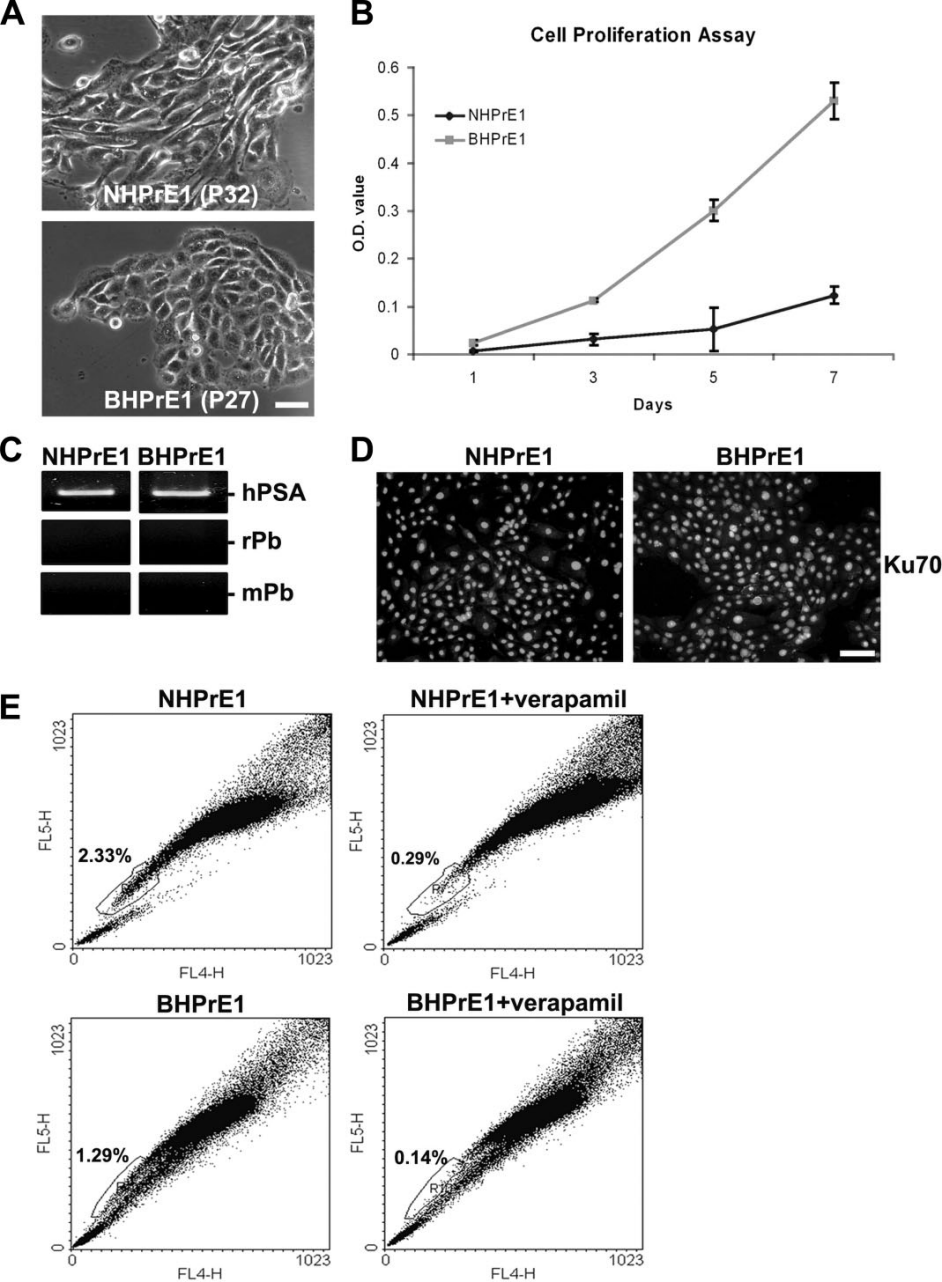
Generation and characterization of human prostate-specific epithelial NHPrE1 and BHPrE1 cells. **A:** Morphology of NHPrE1 and BHPrE1 cells at passages 32 and 27, respectively. Scale bar = 50 μm. **B:** NHPrE1 cells grow more slowly than BHPrE1 cells in a cell proliferation assay. **C:** Polymerase chain reaction detection of hPSA, rPb, and mPb in the genomic DNA confirms the human origin of NHPrE1 and BHPrE1 cells. **D:** Ku70 staining is 100% positive by immunofluorescence in both NHPrE1 and BHPrE1 cells, suggestive of human origin. Scale bar = 50 μm. **E:** Active export of bis-benzimide (Hoechst 33342) identifies a side population of prostate epithelial cells in NHPrE1 and BHPrE1 cells by flow cytometry analysis (outlined), which is reduced upon the addition of verapamil treatment (right), suggestive of a stem cell population. Abbreviations: BHPrE0, primary cultured benign human prostate epithelial cells isolated from a prostate surgical sample; hPSA, human prostate-specific antigen; mPb, mouse Probasin; NHPrE0 (NHP8), primary cultured “normal” human prostate epithelial cells isolated from a donated, 41-year-old, healthy, male prostate; rPb, rat Probasin.

In culture, both NHPrE1 and BHPrE1 cells showed similar prostate epithelial cell morphology, expressing predominantly basal cell-associated markers (Fig. 2A). NHPrE1 cells proliferated somewhat more slowly under basal conditions than BHPrE1 cells (Fig. 2B), and the two lines showed similar cell cycle distribution profiles by FACS analysis compared to their parental NHPrE0 and BHPrE0 cells (supporting information). Using species-specific polymerase chain reaction primers to amplify human PSA (hPSA), rat Probasin, and mouse Probasin, genomic DNA of NHPrE1 and BHPrE1 cells was confirmed as being of human origin (Fig. 2C). Ku70-IF staining (Fig. 2D) with strong nuclear immunoreactivity was strongly positive in all epithelial cells. A side population–FACS assay was used to detect a putative stem cell population in the NHPrE1 and BHPrE1 cell lines, and these data were compared to the parental primary culture cells NHPrE0 and BHPrE0 [28]. The side population numbers for NHPrE1 cells were approximately twice as great as those for BHPrE1. NHPrE1 demonstrated a 2.33% side population, which was reduced to 0.29% with verapamil treatment. BHPrE1 cells showed a 1.29% side population, which was reduced to 0.14% by verapamil treatment (Fig. 2E). The side population numbers for NHPrE0 cells were approximately 0.0%. BHPrE1 cells showed a 0.08% side population, which was reduced to 0.0% by verapamil treatment (supporting information), suggestive of rare stem cell population in primary cultured normal or benign human prostate epithelial cells.

A soft agar colony formation assay was used to assess anchorage-independent growth. No colony formation was detected in either NHPrE1 or BHPrE1 cells in contrast to C4-2B positive control colony formation (supporting information). This finding is consistent with normal or benign cell types. With long-term continuous culture and more than 75 serial passages, both NHPrE1 and BHPrE1 cells maintained the basal epithelial cell type in vitro in the 5% FBS HPrE-conditional medium.

### Phenotypic Analysis of NHPrE1 and BHPrE1 Cells

**Figure 3:**
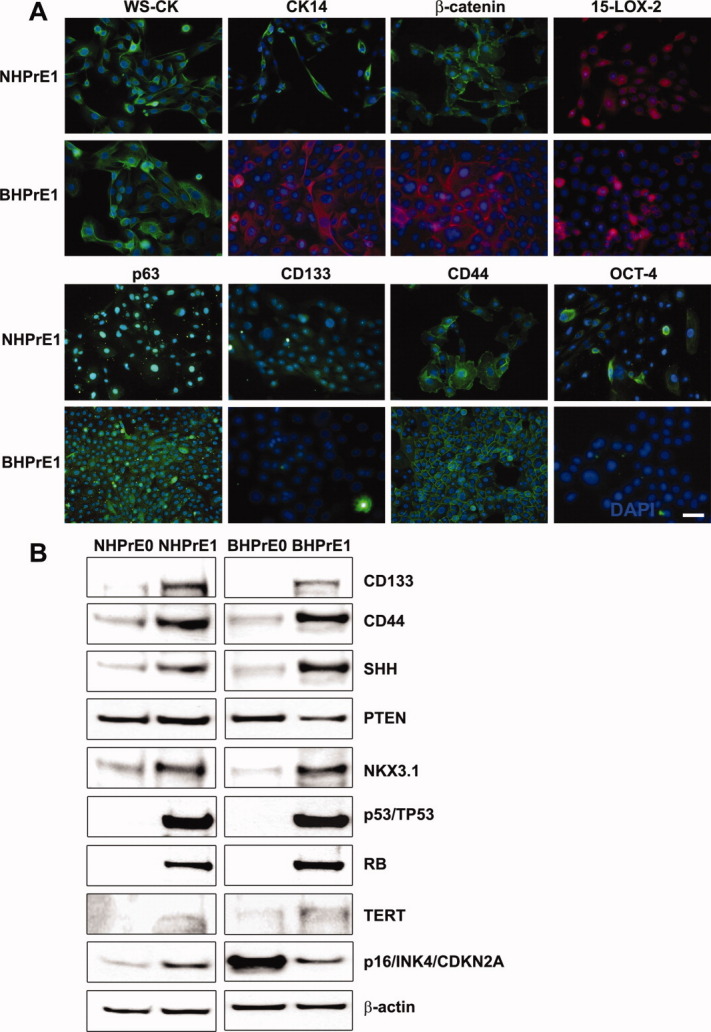
Phenotypic characterization of NHPrE1 and BHPrE1 cells. **A:** Immunofluorescence staining of NHPrE1 and BHPrE1 cells shows 100% immunoreactivity to a WS-CK, confirming the epithelial nature of the cells. NHPrE1 cells show expression of CK14 in most cells, and β-catenin is seen at the membrane interface of adjacent cells while 15-LOX-2 is detected in the cytoplasm. Consistent with the CK14 data, nuclear p63 is present in most cells, as are CD133 and CD44. OCT-4 staining is variable but detectable in most cells. BHPrE1 cells are also epithelial with all cells exhibiting immunoreactivity to WS-CK and many to CK14 but with lower expression of CD133, CD44, and OCT-4. 15-LOX-2 is detected in a minority of cells. Scale bar = 50 μm. **B:** Western blotting shows that CD133, CD44, and PTEN proteins are expressed at elevated levels; however, SHH and RB proteins are expressed at decreased levels in NHPrE1 cells compared to BHPrE1 cells. In contrast, NKX3.1 and the cell cycle–regulating protein p53 are similarly expressed in BHPrE1 and NHPrE1. A moderate level of TERT is shown in both NHPrE1 and BHPrE1 cells compared to NHPrE0 and BHPrE0 cells, respectively. BHPrE1 shows a low level of p16 protein expression compared to BHPrE0. In contrast, NHPrE1 shows enhanced p16 protein compared to NHPrE0. Abbreviations: 15-LOX-2, 15-lipoxygenase-2; BHPrE0, primary cultured benign human prostate epithelial cells isolated from a prostate surgical sample; BHPrE1, a spontaneously immortalized benign human prostate epithelial cell line from BHPrE0; NHPrE0 (NHP8), primary cultured “normal” human prostate epithelial cells isolated from a donated, 41-year-old, healthy, male prostate; NHPrE1: a spontaneously immortalized “normal” human prostate epithelial cell line, that was established from parent NHPrE0 cells; WS-CK, wide-spectrum anti-keratin antibody.

IF staining and Western blotting were used to characterize the NHPrE1 and BHPrE1 cell lines in vitro. Immunophenotyping confirmed both cell lines as human (15-LOX-2), epithelial (WS-CK, CK14, p63, and β-catenin) (Fig. 3A), consistent with the observations described above.

NHPrE1 cells expressed higher levels of the stem cell-associated CD133, CD44, and OCT-4, as well as lower levels of β-catenin and 15-LOX-2 proteins by IF staining compared with BHPrE1 cells (Fig. 3A). This is consistent with a putative progenitor phenotype. In addition, the data generated were compared with those of the parental NHPrE0 and BHPrE0 cells. NHPrE1 cells expressed elevated levels of CD133, CD44, sonic hedgehog (SHH), PTEN (with high AKT and negative P-AKT expression (supporting information)), NKX3.1, p53, and RB by Western blotting, compared with NHPrE0 cells (Fig. 3B). BHPrE1 cells expressed higher levels of CD133, CD44, SHH, NKX3.1, p53, and RB proteins, and a lower level of PTEN protein, by Western blotting, compared with BHPrE0 cells (Fig. 3B). BHPrE1 cells showed similar levels of p53, NKX3.1, AKT, and Cyclin D1 (Fig. 3B and supporting information) as NHPrE1 cells. BHPrE1 cells expressed lower levels of CD133, CD44, PTEN, and OCT-4 (Fig. 3B and supporting information) proteins by Western blotting, compared with NHPrE1 cells. However, BHPrE1 showed higher levels of SHH, RB, C-MYC, p14 (ARF), p21 (WAF1), and the epithelial differentiation markers p63, β-catenin, and E-cadherin, than NHPrE1 cells did (Fig. 3B and supporting information). These data suggested characterization of the BHPrE1 line as a putative intermediate cell type.

TERT protein expression was detected in both NHPrE0 and BHPrE0 cell lines, though at very low levels, along with a moderate level of p16 protein (Fig. 3B), suggesting that spontaneous immortalization may occur by progenitor/stem cell self-renewal and enrichment. Elevated levels of p16 protein seen in BHPrE0 primary cells suggested the possibility of senescence in a subpopulation of cells. A few vimentin-positive NHPrE1 cells were seen with IF staining (data not shown) and confirmed with Western blotting; however, vimentin was not detected in BHPrE1 cells (supporting information). Synaptophysin, a biomarker characteristic of neuroendocrine cells, was detected in a few NHPrE1 cells but was not seen in BHPrE1 cells (data not shown). A few PSA-positive cells were detected with IF staining and a low level of PSA protein expression was seen with Western blotting in BHPrE1 cells, while PSA was not detected in NHPrE1 cells (data not shown). α-Smooth muscle-actin was not detected in either NHPrE1 or BHPrE1 cells (data not shown). No evidence of epithelial to mesenchymal transformation or stromal/mesenchymal cell types was seen even after many passages of continuous culture in vitro.

### Karyotypic and Array-CGH and DIGMAP Analysis

**Figure 4:**
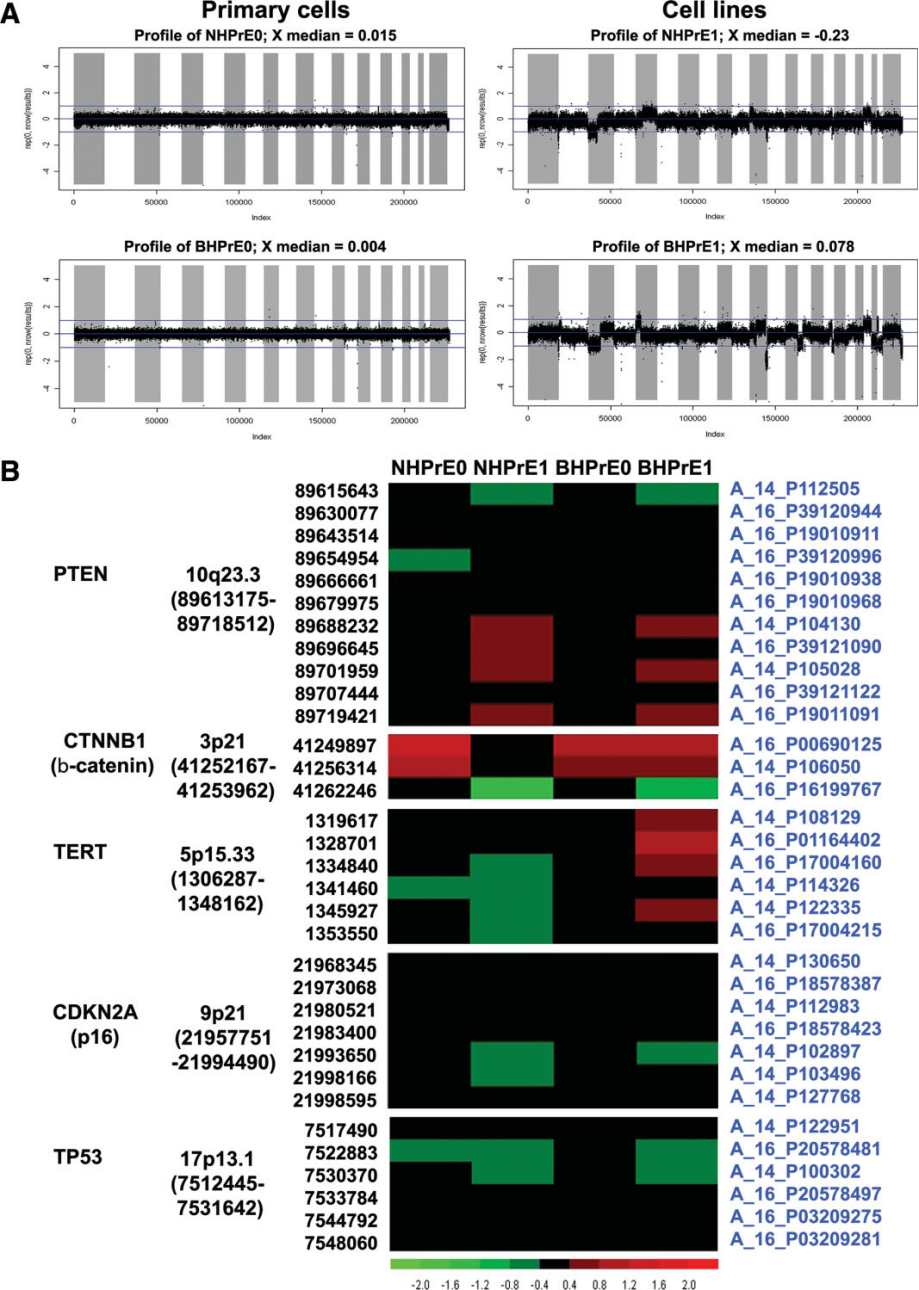
Array–comparative genomic hybridization (array-CGH) and differential gene locus mapping (DIGMAP) analysis of NHPrE0 and BHPrE0 primary cells and derivative NHPrE1 and BHPrE1 cell lines. **A:** Array-CGH of NHPrE0 and BHPrE0 lines reveal a genome with no prominent unbalanced events; karyotypes resemble “normal.” In contrast, the derivative cell lines (BHPrE1 and NHPrE1) show more extensive genomic rearrangements. **B:** Converted DIGMAP data showing alterations in PTEN (amplified in NHPrE1), CTNNB1 (β-catenin amplified in BHPrE1), TERT, CDKN2A (p16), and *TP53*, which facilitate spontaneous immortalization in both lines. All array-CGH data have been deposited at the GEO database (accession number GSE18122). Abbreviations: BHPrE0, primary cultured benign human prostate epithelial cells isolated from a prostate surgical sample; BHPrE1, a spontaneously immortalized benign human prostate epithelial cell line from BHPrE0; NHPrE0 (NHP8), primary cultured “normal” human prostate epithelial cells isolated from a donated, 41-year-old, healthy, male prostate; NHPrE1: a spontaneously immortalized “normal” human prostate epithelial cell line, that was established from parent NHPrE0 cells.

With long-term continuous culture in the serum-supplemented HPrE-conditional medium, NHPrE1 cells showed a near diploid karyotype with a 3n sideline. BHPrE1 cells were near 3n, 65–72 chromosomes with significant chromosomal rearrangements. Array-CGH was used to detect DNA amplification or deletion in genomic samples from NHPrE1 and BHPrE1 cell lines. Global views of the array-CGH results for NHPrE1 and BHPrE1 cell lines, their parental NHPrE0 and BHPrE0 cells were plotted (Fig. 4A). There was an obvious hemizygous deletion of *TP53*, but not WAF1 (p21), Cyclin D1, or RB, in both NHPrE1 and BHPrE1 cells, compared to the array-CGH results for the parental NHPrE0 and BHPrE0 cells (which were apparently genetically intact with minimal chromosomal changes). The copy number changes for chromosome regions were examined using DIGMAP analysis to compare the four cell types (NHPrE0, NHPrE1, BPHPrE0, and BHPrE1) as shown in Figure 4B. The main differential regions between NHPrE1 and BHPrE1 are 17p13.1, 10q23.3, 3p21, 5p15.33, and 9p21. Interestingly, PTEN was amplified in NHPrE1 cells and CTNNB1 (β-catenin) was amplified in BHPrE1 cells (Fig. 4B). These data support the concept that PTEN may be involved in some way in maintaining progenitor cell status [29]. TERT, CDKN2A (p16), and *TP53* status in both cell lines (Fig. 4B) also supported the observed phenotypes during cellular immortalization [19]. An extended DIGMAP analysis of additional progenitor and differentiation markers are listed in supporting information.

### Branching Morphogenesis in 3D Culture by NHPrE1 and BHPrE1 Cells

**Figure 5:**
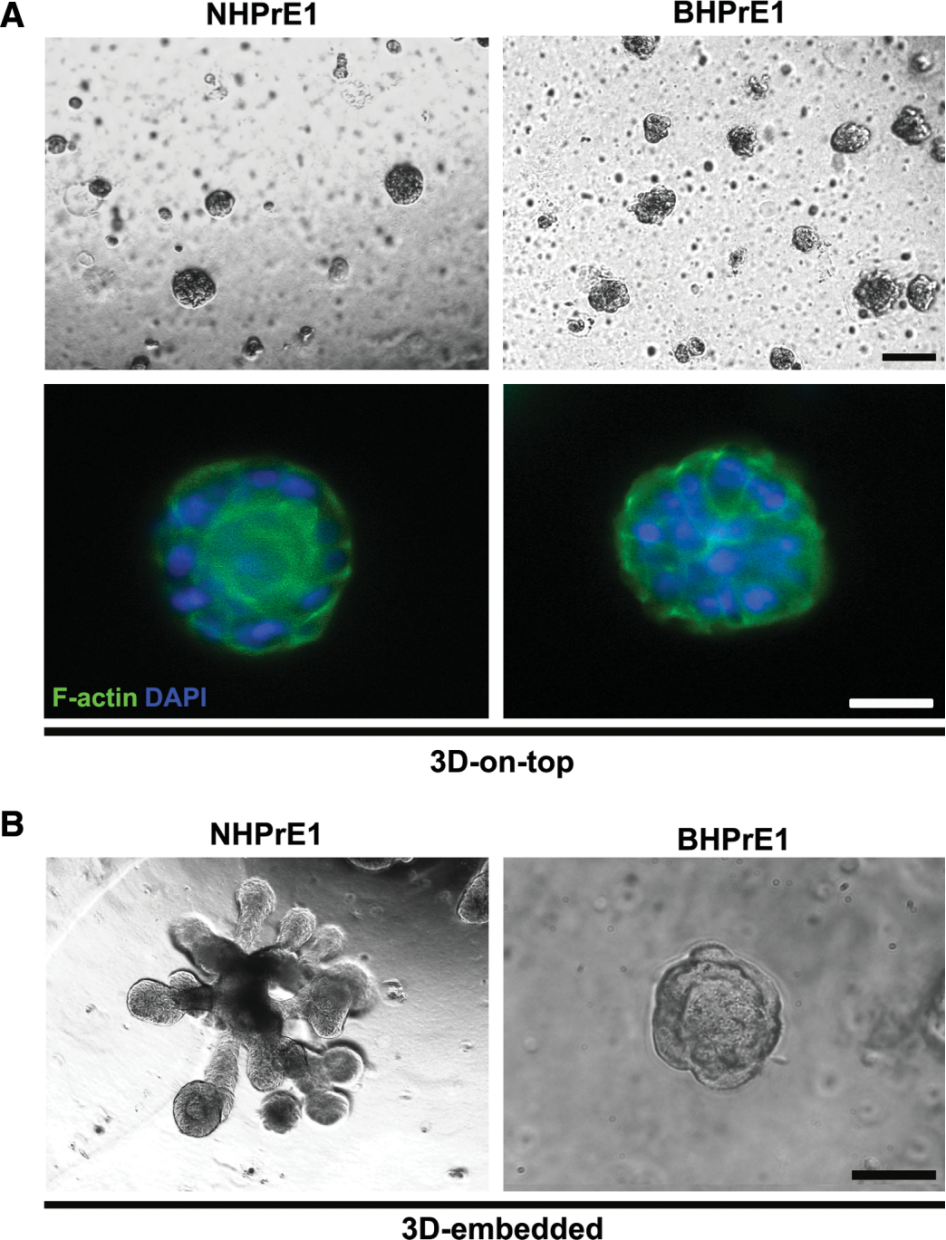
Three-dimensional (3D) culture of NHPrE1 and BHPrE1 cells. **A:** NHPrE1 and BHPrE1 cells form globular structures similar in size and shape after 9 days in a 3D-on-top assay in 2% Matrigel as shown by bright-field microscopy. Confocal imaging (F-actin polymerization shown by phalloidin staining) shows a degree of polarization but no obvious glandular formation in these structures. **B:** NHPrE1 or BHPrE1 cells produce very different structures when completely embedded in Matrigel after 9 days. NHPrE1 can give rise to elegantly branched structures, whereas BHPrE1 cells form globular structures of similar size and shape to those seen in the 3D-on-top assay. Abbreviations: BHPrE1, a spontaneously immortalized benign human prostate epithelial cell line from BHPrE0; NHPrE1: a spontaneously immortalized “normal” human prostate epithelial cell line, that was established from parent NHPrE0 cells.

3D culture is a valuable in vitro model for studying tissue branching, tubulogenesis, and morphogenesis. Tumorigenic cells often fail to appropriately organize and polarize in 3D culture [30]. Therefore, to determine whether NHPrE1 and BHPrE1 cells could organize appropriately in 3D culture conditions, cells were either embedded in 2% Matrigel (3D-on-top) or completely within Matrigel, according to previously established methods [31]. When embedded in 2% Matrigel plus HPrE-conditional medium (3D-on-top assay), polarized acinar structures were formed by both cell lines, as confirmed with F-actin immunolocalization (Fig. 5A). When embedded completely in Matrigel, NHPrE1 cells sometimes initiated highly branched structures, which were never observed using BHPrE1 cells (Fig. 5B), possibly reflecting the significant difference in side population percentage (Fig. 2E).

### Functional Regeneration of Benign Human Prostatic Tissues in Mice Using NHPrE1 and BHPrE1 Cells with Inductive Rat UGM

**Figure 6:**
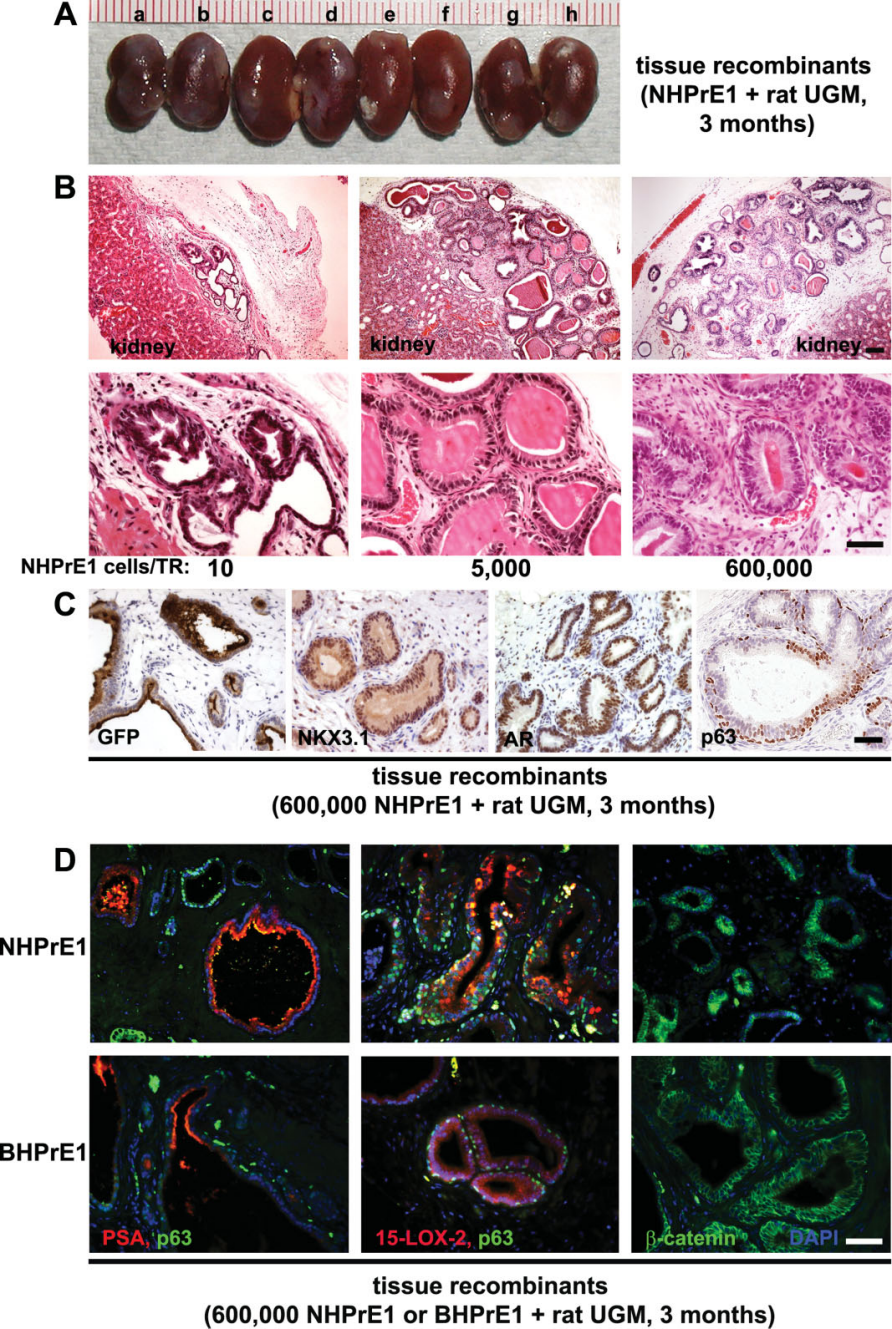
Functional remodeling of benign human prostatic architecture in vivo. **A:** Gross images of tissue recombinants grafted under the sub-renal capsule of SCID mice for three months using different ratios of NHPrE1 epithelial cells {10 (a, b), 5,000 (c, d), 50,000(e, f), 100,000 (g, h)} with rat UGM. **B:** Histology of tissue recombinants made by NHPrE1 plus rat UGM using 10, 5,000 or 600,000 epithelial cells. Note the well differentiated tall columnar secretory cells and expression of secretion into the glandular lumen (position of host kidney noted). Scale bar = 100 μm (low mag.) or 50 μm (high mag.). **C:** Immunohistochemical detection of GFP, NKX3.1, AR and p63 show functionally differentiated prostatic regeneration in tissue recombinants made with NHPrE1-EGFP cells plus rat UGM. NKX3.1 and AR are expressed throughout the luminal epithelium and in some stromal cells, p63 marks the basal epithelium. Scale bar = 50 μm. **D:** Double immunofluorescence staining was used to detect p63 and human prostate specific biomarker PSA or 15-LOX-2 in tissue recombinants made by NHPrE1 or BHPrE1 cells with rat UGM. p63 was confirmed to the basal cells in mature ducts while PSA and 15-LOX-2 were expressed in well differentiated luminal epithelial cells. β-catenin was seen by immunofluorescence to be localized in epithelial cellular membrane in mature NHPrE1- or BHPrE1-tissue recombinants. Scale bar = 50 μm. Abbreviations: BHPrE1, a spontaneously immortalized benign human prostate epithelial cell line from BHPrE0; EGFP, enhanced green fluorescent protein; GFP, green fluorescent protein; NHPrE1: a spontaneously immortalized “normal” human prostate epithelial cell line, that was established from parent NHPrE0 cells; UGM, urogenital sinus mesenchyme.

To investigate morphological and biological features of the NHPrE1 and BHPrE1 cell lines, a tissue recombination-xenografting model was used to reconstruct human prostatic glandular architecture in an immunodeficient SCID mouse model. Tissue recombinants made by mixing human prostate epithelial cell lines with inductive rat UGM in type I collagen were implanted under the renal capsule of testosterone-supplemented male SCID mice (Fig. 6A). A series of different numbers of NHPrE1 or BHPrE1 epithelial cells (10, 5,000, 50,000, 100,000, 200,000, 400,000, 600,000, and 800,000) to a single rat UGM (making four small pieces) were tested to empirically determine growth of tissue recombinants in vivo (Table 1).

**Table 1:** Tissue recombinants made by different ratios of epithelial cells with rat urogenital sinus mesenchyme

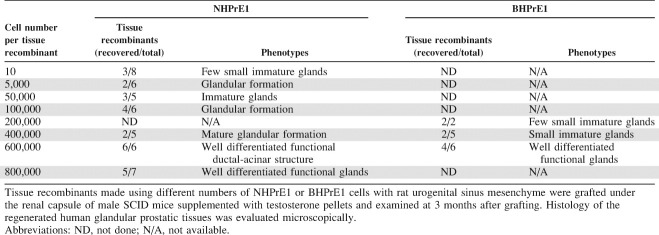

A minimum number of 10 NHPrE1 cells gave rise to small immature glands within 3 months (Fig. 6B). In contrast, a minimum of 200,000 BHPrE1 cells was required to generate similar structures. A ratio of 600,000 NHPrE1 or BHPrE1 cells to the mesenchymal cells derived from a single rat UGM gave rise to the best functional remodeling of benign human prostatic glandular tissues in mice (Fig. 6B). This ratio of epithelial cells/UGM was standardized for most experiments (Fig. 6C, 6D).

Histologically, tissue recombinants of NHPrE1 cells gave rise to mature, well-differentiated glandular epithelium with organization closely resembling the human prostate after 3 months. Reconstituted glandular tissues showed the typical two layers of luminal and basal cells, and a glandular lumen filled with secretory fluid (Fig. 6B). Epithelial cell origin was confirmed using GFP-tagging, with subsequent IHC staining of NHPrE1-EGFP cells (Fig. 6C) and BHPrE1-EGFP (data not shown). These findings were also confirmed by direct observation of GFP by IF (data not shown). AR and NKX3.1 proteins were weakly expressed in the basal cells but strongly expressed in the 100% luminal cells (Fig. 6C). p63 protein was expressed in the basal cells (Fig. 6C). By double IF staining, human prostate-specific biomarkers PSA and 15-LOX-2 proteins were expressed in the luminal cells of both NHPrE1- and BHPrE1-prostatic tissue recombinants, while p63 protein was found in the basal cells (Fig. 6D). β-Catenin (Fig. 6D) and E-cadherin (data not shown) were strongly expressed at the cellular membrane.

Tissue recombinants made with BHPrE1 cells showed a phenotype consistent with mature prostatic acini by H&E and PAS (supporting information) by 3 months after grafting. Histology and IF and IHC staining, which showed biomarker expression patterns in the reconstituted glandular tissues, resembled those seen both in the source of human prostate tissue (supporting information) and in recombinants made with BHPrE0 (Fig. 1A, 1B), NHPrE1 (Fig. 6B–6D), and NHPrE0 cells [19].

Consistent with many previous studies, control grafts of epithelial cells without rat UGM did not show any glandular organization and also did not form any appreciable masses in mice (data not shown), which confirms the lack of malignant potential in NHPrE1 and BHPrE1 cell lines.

These results, using human prostate-specific epithelial cell lines NHPrE1, a progenitor cell type with characteristics of CD133^high^/CD44^high^/OCT4^high^/PTEN^high^, and BHPrE1, an intermediate cell type with characteristics of p63^high^/p53^high^/p21(WAF1)^high^/RB^high^, mixed with inductive rat UGM demonstrate in an in vivo mouse model that mature human benign prostatic epithelial tissues are able to be functionally regenerated from immortalized lines, with expression of a range of mature prostatic markers.

## DISCUSSION

We established two adult, non-tumorigenic human prostatic epithelial cell lines: NHPrE1, which retains progenitor characteristics, and BHPrE1, which displays epithelial intermediate/transit amplifying cell characteristics. These cell lines were generated without external DNA or viral modification. Cells cultured in serum-supplemented HPrE-conditional medium in vitro were subsequently able to recapitulate key developmental events in a tissue recombination-xenograft model, and to regenerate well-differentiated secretory prostatic tissues, expressing human prostatic biomarkers including PSA and 15-LOX-2 and the prostate-associated transcription factor NKX3.1. The NHPrE1 and BHPrE1 cell lines will be valuable tools to further develop our understanding of both cellular lineages and the molecular mechanisms underlying human prostatic development and pathogenesis.

### Possible Mechanisms of Spontaneous Immortalization

Many methods have been used to immortalize cells. The use of viral oncogenes is efficient but results both in significant alterations to the cell cycle machinery and in the inactivation of key pathways such as p53 and RB, making the cells susceptible to genomic instability and malignant transformation. Other approaches have included the overexpression of human TERT or knockdown of p16, which has some advantages in terms of reducing cellular changes [19,32] but, like all model systems, also retains some problems (reviewed in [32,33]). In this study, a spontaneous immortalization that allows cells to come through crisis and then derive immortalized lines has been used. The advantages of this approach include limiting genetic damage to key cell cycle checkpoints and allowing for the derivation of cells that are able to recapitulate key aspects of prostatic biology when appropriately stimulated by inductive mesenchyme. Negative results from the soft agar colony formation assay and the benign nature of both NHPrE1 and BHPrE1 cell lines in the tissue recombination assays confirmed the lack of malignant transformation. Telomerase expression was found to be elevated in both NHPrE1 and BHPrE1 cells compared with their parental primary culture cells, while p16 protein expression was decreased in BHPrE1 cells, potentially representing routes contributing to their immortalization. High levels of p16 protein expression in BHPrE0 suggested possible senescence occurring in cell culture. In contrast, p16 protein was elevated in NHPrE1 cells compared to NHPrE0 cells, suggestive of progenitor/stem cellular self-renewal behaviors.

### Cell Lineage Commitments in Prostatic Organogenesis and Morphogenesis

The development of the prostate results from androgen-driven mesenchymal-epithelial interactions [2]. Some key details of this process remain unclear. These include documenting the precise nature of the growth and transcription factors that mediate these interactions, an area that has been widely investigated and in which much progress has been made [34–42]. A less well-understood aspect has been the nature of the epithelial cells involved in the development process. It has long been supposed that a stem/progenitor cell hierarchy exists within the prostate [5,43]. This concept enjoys some experimental support [14]. The precise nature of the cells concerned has not been clarified; however, expression of a number of markers, including CD44, SCA1, and OCT4, has been considered to be associated with “stemness” [44,45]. In this study, we present two new cell lines, NHPrE1 and BHPrE1. The NHPrE1 cell line, which is shown by chromosomal analysis to be a mixed population, was provisionally classified as including a self-renewing progenitor population (possibly the identified side population) on the basis of expression of a set of candidate stem and basal cell markers in vitro. The BHPrE1 cell line, which is also a genetically mixed population, behaves in bulk like an intermediate or transit-amplifying cell type due to its expression of previously characterized markers including p63, NKX3.1, β-catenin, and E-cadherin [46,47].

3D culture can be used to discriminate between normal and malignant cells [31]. NHPrE1 and BHPrE1 cells were both shown to be capable of forming polarized ductal or acinar structures in 3D Matrigel surface assays, and NHPrE1 cells were uniquely able to undergo branching in 3D Matrigel embedded assays, possibly suggesting a high ratio of stem/progenitor-like cells. BHPrE1 cells were not able to make branching structures in 3D cultures, at least under these conditions, consistent with a high ratio of intermediate cell type.

In tissue recombinants, made by mixing different ratios of epithelial cells with a fixed quantity of rat UGM tissue, functional regeneration of mature human prostate glandular structure was found to depend on the number and nature of implanted human prostate epithelial cells. These experiments support the standard model of an intermediate/transit-amplifying population that can recapitulate prostatic development. The BHPrE1 cells were able to differentiate into prostatic epithelial tissue containing both basal and luminal epithelial cells. However, it was noted that the more progenitor-like NHPrE1 population was able to perform this task more efficiently and with a lower number of cells. As few as 10 NHPrE1 cells could give rise to glandular tissue, while the minimum number of BHPrE1 cells needed for the same task was 200,000. This may reflect characteristics of the bulk population of cells or, alternatively, the relatively elevated number of “stem-like” cells in the NHPrE1 population. The expression of stem cell–associated markers, notably CD133, OCT-4, and CD44 at elevated levels in NHPrE1 cells compared to BHPrE1, was consistent with the concept of these cells representing or containing a “progenitor” population.

### Conclusion

Our data suggest that functional regeneration of benign prostate glandular architecture is more efficient when initiated from a population representing or containing a major subpopulation of putative progenitor cells rather than of intermediate/transit amplifying epithelial cells. We demonstrate that immortalized human prostatic epithelial cell lines are able to recapitulate prostatic development. One of the major strengths of these lines is their ability to form appropriate ductal-acinar architecture containing epithelial subpopulations with appropriate expression of markers including androgen receptor, NKX3.1, PSA, and 15-LOX-2. While there are genomic alterations in the lines presented here associated with crisis and subsequent spontaneous immortalization, these cells are behaviorally benign as assessed with histopathological criteria. As such, these cell lines represent potentially useful models in which to start to investigate mechanisms associated with both benign and malignant disease.
