# Supplementary material for: Functional Remodeling of Benign Human Prostatic Tissues In Vivo by Spontaneously Immortalized Progenitor and Intermediate Cells
Source: Stem Cells. 2010 Jan 28;28(2):344–56. doi: 10.1002/stem.284 (PMC2962907; doi:10.1002/stem.284)
Supplement: Supplementary file 6 [file stem0028-0344-SD6.doc]

Supplemental Table 1. Antibody sources and dilutions used for analysis.

| **Protein** | **Company (Cat#)** | **Dilution** | **Functions** |
| --- | --- | --- | --- |
| Ku70 | Abcam (ab10858-50) | 1:100 (IF) | Human DNA repair protein |
| SHH | Cell Signaling (2207) | 1:500 (WB) | Stem cell marker |
| CD133 | Abcam (ab16518) | 1:200 (IF/WB) | Stem cell marker |
| CD44 | Santa Cruz (sc-7297) | 1:200 (IF/WB) | Stem cell marker |
| OCT-4 | Cell Signaling (2750) | 1:200 (IF/WB) | Stem cell marker |
| PTEN | Cell Signaling (9559) | 1:500 (WB) | Cell survival; stem cell |
| Phospho-AKT  (P-AKT) | Cell Signaling (9271) | 1:500 (WB) | Cell survival |
| AKT | Cell Signaling (4691) | 1:1000 (WB) | Cell survival |
| TERT | Santa Cruz (sc-7212) | 1:200 (WB) | Immortalization |
| Vimentin | Sigma (V5255) | 1:500 (WB)  1:1000 (IF) | Intermediate filament |
| E-cadherin | BD Biosciences (610181) | 1:1000 (WB)  1:500 (IF) | Cellular adhesion |
| β-catenin | BD Biosciences (610154) | 1:500 (WB)  1:100 (IF) | Cellular adhesion |
| Rb | BD Biosciences (554136) | 1:500 (WB) | Cell cycle control |
| p16 | Santa Cruz (sc-56330) | 1:200 (WB) | Cell cycle control |
| p53 | Santa Cruz (sc-126) | 1:1500 (WB) | Cell cycle control |
| p63 | Santa Cruz (sc-8431)  (sc-8609) | 1:500 (WB)  1:200 (IHC/IF) | Cell cycle control and benign prostate basal cell marker |
| PSA | Dako (A0562) | 1:200 (WB)  1:100 (IF) | Human prostatic differentiation |
| 15-LOX-2 | Cayman (10004454) | 1:200 (WB)  1:100 (IF) | Metabolism, human prostate marker |
| AR | Santa Cruz (sc-816) | 1:500 (WB)  1:1000 (IHC) | Differentiation |
| NKX3.1 | Zymed (35-9700) | 1:250 (WB)  1:250 (IHC/IF) | Prostate-specific transcription factor |
| WS-CK | Dako (Z0622) | 1:100 (IF) | Epithelial marker |
| CK14  (LL001) | Dr. EB Lane, (University of Dundee, Scotland). | 1:10 ( IHC/IF) | Epithelial marker |
| CK18  (LE61) | Dr. EB Lane, (University of Dundee, Scotland). | 1:10 ( IHC/IF) | Epithelial marker |
| α-SM-actin | Sigma (A2547) | 1:1000 (IHC) | Smooth muscle marker |
| GFP | Santa Cruz (sc-8334) | 1:250 (IHC) | Molecular tagging tracker |
| β-actin | Sigma (A5441) | 1:1500 (WB) | Housekeeping |
